# A genotyping by sequencing approach can disclose *Apis mellifera* population genomic information contained in honey environmental DNA

**DOI:** 10.1038/s41598-022-24101-z

**Published:** 2022-11-15

**Authors:** Samuele Bovo, Valerio Joe Utzeri, Anisa Ribani, Valeria Taurisano, Giuseppina Schiavo, Luca Fontanesi

**Affiliations:** Division of Animal Sciences, Department of Agricultural and Food Sciences, University of Bologna, Viale Giuseppe Fanin 46, 40127 Bologna, Italy

**Keywords:** Genome, Genomics, Population genetics, Sequencing, Genomic analysis, High-throughput screening, Sequencing, Computational biology and bioinformatics

## Abstract

Awareness has been raised over the last years on the genetic integrity of autochthonous honey bee subspecies. Genomic tools available in *Apis mellifera* can make it possible to measure this information by targeting individual honey bee DNA. Honey contains DNA traces from all organisms that contributed or were involved in its production steps, including the honey bees of the colony. In this study, we designed and tested a genotyping by sequencing (GBS) assay to analyse single nucleotide polymorphisms (SNPs) of *A. mellifera* nuclear genome using environmental DNA extracted from honey. A total of 121 SNPs (97 SNPs informative for honey bee subspecies identification and 24 SNPs associated with relevant traits of the colonies) were used in the assay to genotype honey DNA, which derives from thousands of honey bees. Results were integrated with information derived from previous studies and whole genome resequencing datasets. This GBS method is highly reliable in estimating honey bee SNP allele frequencies of the whole colony from which the honey derived. This assay can be used to identify the honey bee subspecies of the colony that produced the honey and, in turn, to authenticate the entomological origin of the honey.

## Introduction

*Apis mellifera* L., the Western honey bee, is the most relevant managed pollinator species. It comprises about 30 subspecies, originally described based on their geographic distribution and morphometric characteristics that have been subsequently integrated with DNA-derived information^1–4^. These subspecies have been grouped in four or five evolutionary lineages^1,3–6^: lineage A, the African lineage; lineage C, of South-Eastern European populations; lineage M, of Northern and Western European honey bees; lineage O, of Middle-East populations; and the recently proposed lineage Y, of North-Eastern African populations.

Over the last decades, the natural distribution range and genetic integrity of many European honey bee subspecies have been largely modified by the combined result of several anthropogenic actions, including large-scale commercial trade of queens, non-traditional queen selection programs and transhumance^7–9^. These actions, in many cases, are caused by the necessity of the beekeeping sector to cope with more global threats. For example, the effect of adverse climate conditions on nectar availability incentives migratory beekeeping^10,11^. The extensive use of harmful pesticides for the bees and the related increased sensitivity to parasites and pathogens are indicated as some of the main causes of the global decline of the bees and colony losses which, in turn, incentive the use of non-autochthonous genetic stocks to replace dead colonies if local queen production programs cannot satisfy the requests of local beekeepers^12–14^. The general effect is that several locally adapted honey bee populations or ecotypes are at risk due to admixture and crossbreeding with non-autochthonous subspecies and genetic pools. The consequence can be the potential loss of local adaptation traits that are considered critical for the long-term sustainability of beekeeping activities and pollination services in the agroecological environments and production systems^15–17^. Awareness on these issues has been recently raised in several European countries where conservation programs of autochthonous honey bee genetic resources have been started or envisaged^7,8,18–25^.

Monitoring and conservation programs of autochthonous honey bee subspecies rely on the possibility to certify their genetic integrity. Variability at the nuclear genome level can provide useful information. To this aim, single nucleotide polymorphism (SNP) panels have been designed starting from whole genome resequencing datasets produced from different honey bee populations, and informative markers that can discriminate honey bee subspecies have been identified^26–32^. A few SNP panels have been also designed and used for several other applications in honey bee population genetic studies^33–37^. For example, genome-wide association analysis for varroa-specific defence behaviour in honey bees has been carried out using a SNP panel^38^. A few genome-wide association studies for other relevant traits have been also carried out using whole genome resequencing data^39,40^. All these studies have been carried out by genotyping individual honey bees which could be a quite relevant limit, in terms of genotyping cost, to obtaining whole colony population genomic information.

Honey contains environmental DNA (eDNA) traces derived from all organisms that directly or indirectly contribute to its production or that have been part of the production niche and environment from which this matrix is obtained^41,42^. Therefore, honey also contains DNA traces of thousands of honey bees of the colony that produced it. We recently used these traces to set up diagnostic methods to detect lineage specific mitochondrial DNA (mtDNA) haplotypes that could be informative to authenticate the entomological origin of the honey^43,44^. Using assays that can discriminate the major mtDNA lineages, we also reported an updated distribution map of these honey bee mtDNA lineages over all Italy^45^. As commercial honey is usually obtained from more than one colony or even more than one apiary, different mtDNA lineages can be detected from the same honey sample, providing approximate population genetic information useful to estimate the diffusion and frequency of honey bee mitotypes^45^. Honey eDNA has been also explored by using next generation sequencing (NGS) approaches to identify the complementary sex determiner (*csd*) alleles present in the colonies from which the honey was produced^46,47^. The prevalent queen alleles and the frequency of other alleles could be inferred or estimated with this approach^46,47^. Again, using honey eDNA and shotgun sequencing produced by deep NGS, it has been also possible to estimate allele frequencies of honey bee nuclear genome SNPs and assign the colonies to the corresponding honey bee subspecies based on the genetic fingerprint of the honey bees retrieved in this way^42^. Therefore, honey eDNA can be exploited to recover honey bee population genomic information useful to detect genomic parameters at the colony level and, if the honey derives from more than one colony, even at a higher level. It is worth to mention that the mining of deep NGS data, however, requires specifically designed bioinformatic pipelines and is computationally quite demanding.

In this methodological study, we designed and tested a genotyping by sequencing (GBS) assay to simplify the genotyping of honey bee nuclear genome markers useful to provide population genomic information directly from honey eDNA. Genotyping information obtained from honey eDNA was compared with genotyping results obtained from honey bee DNA. The composition of the SNP panel can be modulated (i) to answer questions on the genetic origin of the honey bees and thus to certify the honey bee subspecies that produced the tested honey samples and (ii) to indirectly obtain information on genetic characteristics relevant for the beekeeping activities associated to some markers.

## Methods

### Honey bee and honey samples

A total of 61 specimens were used for the subsequent genomic analyses (Table 1). These specimens were obtained from a few Italian regions (Emilia-Romagna, Liguria and Sicily) and derived from *A. m. ligustica*, *A. m. mellifera* (that experienced some undefined introgression from *A. m. ligustica*) and *A. m. siciliana*, respectively. Specimens included: (i) four honey bee female larvae of *A. m. ligustica* for individual genotyping, each derived from a different colony, (ii) three groups of 35 adult worker honey bees of *A. m. ligustica* for genotyping of DNA pools (see below for details), each group derived from the same colony from which one honeycomb was collected (see below for details), (iii) honey samples obtained each from one honeycomb (thereafter named honeycomb samples), derived from 32 different *A. m. ligustica* colonies, one *A. m. mellifera* colony and one *A. m. siciliana* colony and (iv) undifferentiated honey samples (i.e. each obtained from several colonies belonging to the same apiary), produced from *A. m. mellifera* (two samples) and *A. m. siciliana* (10 samples). *A. m. ligustica* specimens were obtained from colonies raised in the Emilia-Romagna region (North of Italy), *A. m. mellifera* honeycomb and honey samples were obtained from apiaries in the Liguria region (North-West of Italy) and *A. m. siciliana* honeycomb and honey samples were obtained from apiaries in Sicily. Honey was produced in the years 2020 and 2021. Duplicate samples were also included in the study (Table 1). More details on all these specimens are given in Supplementary Table S1. In addition, honeycomb samples and honey samples have been also previously analysed to identify the honey bee mtDNA lineage using the protocols described by Utzeri et al.^43,44^. *A. m. ligustica* colonies from which larvae, worker bees and honeycomb samples were retrieved were confirmed to belong to this subspecies using a classical morphometric method^3,48^.

**Table 1:** Summary of the biological specimens (honey bee and honey samples) analysed in this study.

Specimens	No. of unique + duplicated samples^1^		
	*A. m. ligustica*	*A. m. mellifera*	*A. m. siciliana*
Honey bee (single larvae)	4	0	0
Honey bee DNA pools^2^	3 + 2	0	0
Honeycomb samples	32 + 4	1	1
Honey^3^	0	2	10 + 2
Total no. of unique samples	39	3	11
Total no. of samples	45	3	13

^1^Unique samples + duplicate samples: duplicates were from DNA pools of *A. m. ligustica* (no. = 2), honeycomb honey samples of *A. m. ligustica* (no. = 4), and honey samples of *A. m. siciliana* (no. = 2).

^2^Each DNA pool was constituted by DNA obtained from 35 different honey bees sampled from the same colony at the same time (see the text for details).

^3^Undifferentiated honey samples derived from several colonies of the same apiary.

### DNA extraction and mitochondrial DNA analysis from honey DNA

All honeycombs were separately collected and then managed to avoid any contacts with other honeycombs. This avoided any contamination between honey produced by different colonies. All subsequent procedures were carried out to avoid the same problem, including the use of sterilized apparatus and tubes and molecular grade reagents. Honey was separated from the honeycomb using a gravimetric method at room temperature that included a filtering step to eliminate residual materials. DNA extraction from honey was carried out following the protocol described by Utzeri et al.^49,50^, which included preparatory steps that eliminated the sugars after a series of centrifugations and washings from the pelleted materials and subsequent steps that isolated the DNA contained in the retrieved pellets. This latest part of the protocol included a resuspension of the pellet in 0.5 mL of ultrapure water and added to 1 mL of CTAB extraction buffer [2% (w/v) cetyltrimethylammoniumbromide; 1.4 M NaCl; 100 mM Tris–HCl; 20 mM EDTA; pH 8], prepared with 5 μL of RNase A solution (10 mg/mL) and 30 μL of proteinase K (20 mg/mL), and then incubated at 65 °C for 90 min with gentle mixing. The tube was cooled to room temperature and centrifuged for 10 min at 16,000×*g*. A total of 700 μL of supernatant was transferred to another tube containing 500 μL of chloroform/isoamyl alcohol (24:1), vortexed for 30 s and then centrifuged at 16,000×*g* for 15 min at room temperature. The supernatant was transferred to a 1.5 mL tube and the DNA was precipitated with isopropanol and then ethanol/water 70:30 v/v, following a standard protocol. The DNA pellet was rehydrated with 30 μL of sterile H_2_O and stored at − 20 °C until PCR analyses.

DNA extraction from larvae and worker bees was carried out using the Wizard Genomic DNA Purification Kit (Promega, Madison, MI, USA) following the manufacturer’s instructions for animal tissues. Extracted DNA was evaluated by electrophoresis on TBE 1X 1% agarose gels stained with 1X GelRed Nucleic Acid Gel Stain (Biotium Inc., Hayward, CA, USA) and quantified using a Nanophotometer P-330 instrument (Implen GmbH, München, Germany). Five DNA samples were obtained by pooling together equimolar DNA concentrations of 35 worker bees each.

DNA extracted from honeycomb samples and honey samples has been used to identify the honey bee mtDNA lineage of the bees that produced these food matrices. For these analyses the protocols described by Utzeri et al.^43,44^ were applied. This information was used to complement nuclear genome SNP information derived by the genotyping by sequencing analyses described below.

### Genotyping by sequencing

A total of 121 biallelic SNPs (Supplementary Table S2) of the *Apis mellifera* nuclear genome were selected for the genotyping by sequencing (GBS) analysis using the AgriSeq platform of Thermo Fisher Scientific Inc. (Waltham, MA, USA). SNPs included in the panel (i) can be useful to differentiate the honey bee subspecies and evolutionary lineages (97 ancestry informative SNPs;^27^), (ii) are associated with calmness (3 SNPs;^51^) and gentleness (3 SNPs;^51^) of honey bees and (iii) are associated with resistance to *Varroa destructor* (18 SNPs;^38,40,52^). Based on the original positional information provided in the referred manuscripts (see above), location of the SNPs on the latest version of the *Apis mellifera* reference genome Amel_HAv3.1 (GCF_003254395.2; *Apis mellifera* Strain: DH4) was retrieved by means of flanking DNA sequences of SNPs mapped via BLAST + v.2.7.1, as described by Bovo et al.^42^. The AgriSeq GBS pipeline and analytical flow was based on a multiplexed PCR chemistry (Thermo Fisher Scientific Inc.). Extracted DNA was quantified using the Quant-iT Ds DNA Assay (Thermo Fisher Scientific Inc.). Samples were normalized to a final concentration of 3.3 ng/μL. Libraries were constructed using the AgriSeq™ HTS Library kit (Thermo Fisher Scientific Inc.) and sequenced using the Ion 540 Chef kit and the Ion 540 Chip on the Ion S5 platform (Thermo Fisher Scientific Inc.).

### Sequencing data processing and data analysis

The Ion Torrent Suite Software v.5.12 (Thermo Fisher Scientific Inc.) was used for the analysis of sequencing data. Reads were mapped on the *Apis mellifera* Amel_HAv3.1 genome version using the Ion Torrent Mapping Alignment Program (TMAP) v.5.12.28 (Thermo Fisher Scientific Inc.). Variants were called within each sample with the Ion Torrent Variant Caller (TVC) v.5.12–28 (Thermo Fisher Scientific Inc.) using default parameters. VCF files were joint and allele frequencies (AF) of the SNPs were estimated in each sample of Table 1, considering the number of reads supporting the reference (REF) and alternative (ALT) alleles.

To obtain a global evaluation of the honey bee subspecies and evolutionary lineages, we also retrieved the genotype of the 97 ancestry informative markers in 161 additional publicly available whole genome sequencing (WGS) datasets produced from a single honey bee (Supplementary Table S3), downloaded from the European Nucleotide Archive (ENA; https://www.ebi.ac.uk/ena/), and belonging to the following subspecies: 68 from *A. m. capensis* (lineage A), 22 from *A. m. carnica* (lineage C), 9 from *A. m. caucasica* (lineage O), 10 from *A. m. ligustica* (lineage C), 24 from *A. m. mellifera* (lineage M) and 28 from *A. m. scutellata* (lineage A). Sequencing reads derived from these datasets were mapped on the Amel_HAv3.1 reference genome using the BWA-MEM algorithm v.0.7.17^53^ and genotypes were called with BCFtools v.1.15.1^54^. Moreover, data analysis included the genotype information of the same 97 informative SNPs from 450 individual honey bee samples (Supplementary Table S3) reported by Henriques et al.^29^: 10 samples of *A. m. ligustica,* 406 samples of *A. m. mellifera*, 34 samples of *A. m. carnica*. A final matrix of size 672 samples × 97 SNPs, storing the information of the frequency of the alternative allele (ranging from 0 to 100% for honey or DNA pools of honey bees; 0%, 50% or 100% for single honey bee larvae), was obtained and subjected to a Multidimensional Scaling (MDS) analysis, as implemented in R v.3.6.1, as previously described^42^.

## Results

### General statistics of the genotyping by sequencing data

Genotyping by sequencing (GBS) analyses of the 61 samples, which included DNA from single honey bee larvae, DNA pools of honey bees and DNA from honey samples from different sources (Table 1 and Supplementary Table S1), produced a total of 52.6 million (M) sequencing reads. These reads were used to obtain genotype information of 121 bi-allelic SNPs of the *Apis mellifera* nuclear genome. On average, 0.86 M reads/sample was obtained, with a median value equal to 0.16 M and a standard deviation (s.d.) equal to 1.27 M, pointing out a high variability due to the type and quality of the DNA extracted from the different sample matrices. Sequencing statistics are shown in Table 2. Details are given in Supplementary Table S1. Depth of sequencing (DP) of the 121 DNA markers (Supplementary Table S1) was quite variable and matched the variability of the number of sequenced reads.

**Table 2:** Sequencing data summaries of the number of reads and depth of sequencing.

Specimen	Mean	s.d.	Median	Min	Max
**Number of reads**^**1**^					
Honey	0.14	0.13	0.09	0.02	0.44
Honeycomb	0.69	1.05	0.15	0.01	4.07
DNA pools	2.19	0.55	2.20	1.58	3.13
Single larvae	3.32	1.89	2.52	1.79	6.45
**Depth of sequencing**^**2**^					
Honey	1.14	1.06	0.69	0.13	3.51
Honeycomb	5.56	8.39	1.14	0.05	32.42
DNA pools	17.48	10.14	17.62	12.70	24.81
Single larvae	26.45	14.73	20.28	14.44	50.78

^1^Data are expressed in millions.

^2^Data are expressed in thousands.

### Sample and SNP call rates

Depth of sequencing was used to estimate call rates. The sample call rate (Supplementary Table S1), defined as the fraction of called SNPs in a sample over the total number of targeted SNPs (no. 121), was quite high, with mean ± s.d. of 98 ± 2% (median = 98%) and > 92% in the full sample set. The SNP call rate (Supplementary Table S2), defined as the fraction of samples in which the SNP was called, was high too, with a mean ± s.d. of 98 ± 8% (median = 100%). A total of 110 DNA markers (91%) was covered by at least one read in all the 61 analysed samples. If we consider DNA extracted directly from individual honey bees as our golden standard (9 sequenced samples: 3 + 2 DNA pools and 4 larvae, Table 1), the whole SNP set (121/121; 100%) was successfully genotyped in all 9 samples (call rate = 100%). Thus, all the amplicons designed to genotype *A. mellifera* nuclear variants can be efficiently amplified and sequenced by the applied GBS approach. As expected, lower efficiency has been obtained when DNA from honey and honeycomb samples was used in the genotyping analyses: in honey samples, 114 SNPs (94%) and in honeycomb samples 110 SNPs (90%) were completely genotyped. In these two matrices, six markers (bee_snp_42, bee_snp_78, bee_snp_79, bee_snp_93, bee_calm_3, and Var_res_21) had a call rate < 90%. Statistics stratified by source matrix are reported in Supplementary Table S2.

In addition to the 121 targeted SNPs, amplicons allowed to detect a total of other 152, 38 and 24 variants in at least 5%, 50% and 90% of the analysed samples, respectively. Novel variants were mainly SNPs (69%) followed by indels (23%) and complex variants (8%). Details are given in Supplementary Table S4. These additional variants were not further investigated in this context, as they might be in high linkage disequilibrium level with the targeted SNPs.

### Evaluation of the genotyping concordance rate

The genotyping concordance rate (also known as degree of agreement)^55^ of the applied GBS method was evaluated by analysing and comparing the results obtained from duplicated samples, derived from the GBS of DNA extracted independently from two aliquots of a given sample. For this evaluation, different matrices were tested for a total of 16 sequenced samples (no. 8 duplicates), including two DNA pools (obtained from honey bee DNA), two honey samples, and four honeycomb samples. Genotyping concordance was evaluated by means of the intra-class correlation coefficient (ICC) applied to the allele frequency (of the alternative allele) vectors reported in Table S5. We avoided the use of simple correlation as two sets of observations, despite having a high correlation, do not necessarily are in good agreement^55^.

Four duplicated samples (50%) had ICC > 0.99, including all honey bee duplicated samples and two out of four honeycomb duplicated samples. A correlation of ~ 0.98 was observed for one additional honeycomb sample. One remaining honeycomb and the other two honey samples had lower ICC, that ranged from 0.90 to 0.97. Inspection of scatter plots (Supplementary Fig. S1) evidenced that those duplicated samples characterized by a low ICC had some SNPs with quite a different estimated allele frequency, including the bee_calm_3 and bee_snp_78 markers already highlighted as problematic (SNP call rate < 90%).

### Correlations between genotyping datasets

**Figure 1:**
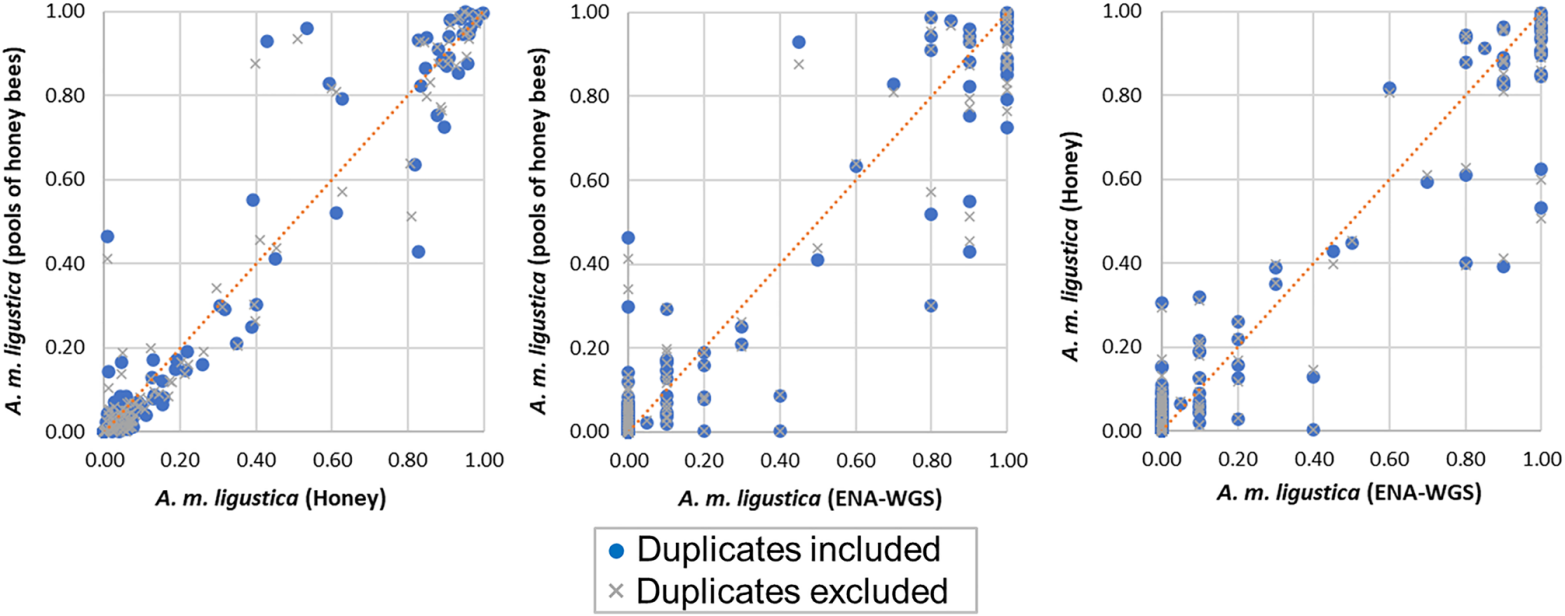
Scatter plots based on frequencies of the alternative allele of the 121 targeted *Apis mellifera* DNA markers. Data are related to the *A. m. ligustica* datasets and samples, for which multiple levels of information were available (larvae data are excluded but provided as Supplementary Fig. S2). Data are presented including/excluding duplicate samples.

Scatter plots based on allele frequencies retrieved for the genotyped SNPs in the GBS SNP panel were obtained to compare *A. m. ligustica* datasets and samples, for which multiple level information was available (i.e. data obtained from DNA of individual honey bee larvae, DNA pools of individual honey bees, DNA from honey extracted from honeycombs and WGS from ENA (Fig. 1 and Supplementary Fig. S2). Analyses considered both the whole sample set (no. = 45) and the subset of unique samples (excluding the duplicates). In the latter case, the samples with the highest call rate or number of reads were retained. Pearson’s (*r*) and Spearman’s rank correlation coefficients (ρ) are reported in Supplementary Tables S6-S8. No substantial differences were evident if duplicate samples were included or not in these comparisons. Pearson’s correlations coefficients were generally high, considering the whole set of analysed SNPs (121 SNPs) or only the 97 ancestry informative SNPs or the other 24 selected SNPs and ranged from 0.87 to 0.97, whereas Spearman’s rank correlation coefficients ranged from 0.76 to 0.99. Low correlations were obtained when the analysis was based on results from larvae, as only four samples are not sufficient to obtain a reliable estimation of allele frequencies (Supplementary Fig. S2).

The three honeycomb samples, for which a DNA pool from honey bees derived from the same colony was analysed, showed a genotyping correlation > 0.94, suggesting a very good approximation of the population genomic data of the colony retrieved from honey DNA.

### Multidimensional scaling analysis from ancestral informative SNPs and mtDNA information on honey samples

Multidimensional scaling (MDS) plots were obtained by including genotyping results of the 97 ancestral informative SNPs obtained from (i) all 61 honey bee and honey derived samples analysed with the GBS assay (Table 1), (ii) the 450 honey bee samples genotyped by Henriques et al.^29^ (Supplementary Table S3) and (iii) the 161 WGS datasets (corresponding to *A. m. capensis, A. m. carnica*, *A. m. caucasica*, *A. m. ligustica*, *A. m. mellifera*, *A. m. scutellata*) and retrieved from a public repository (Supplementary Table S3). The matrix used in the MDS analysis is reported in Supplementary Table S9.

**Figure 2:**
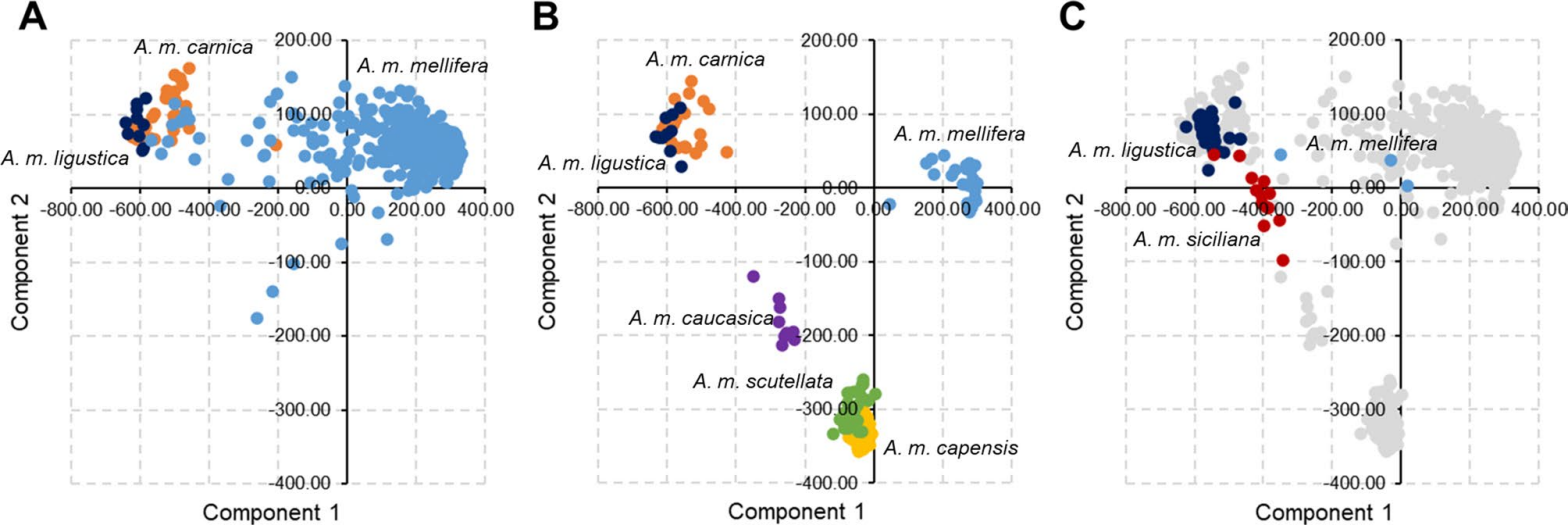
Multidimensional scaling plots (MDS) of the 672 samples combining genotyping by sequencing (GBS) results obtained from specimens analysed in this study and from data retrieved from public sources. The analyses are based on the 97 targeted ancestry informative SNPs. The first two components are presented. Each dot represents a sample. (**A**) Results are from samples and data reported by Henriques et al.^29^. (**B**) Results are from datasets retrieved from ENA. (**C**) Honey, honeycomb and honey bee samples analysed with the described GBS panel. In the background (grey spots), results from the sets A and B.

Figure 2a shows the sample distribution of the data provided by Henriques et al.^29^ and obtained with the same 97 SNPs included in the GBS panel. As expected, two main clusters separating the lineages C (*A. m. carnica* and *A. m. ligustica*) and M (*A. m. mellifera*) emerged. Figure 2b shows the samples distribution of WGS data obtained using the same 97 SNPs that, for samples belonging to the lineages C, perfectly overlapped those presented in Fig. 2a. Moreover, two additional clusters emerged, pointing out the possibility to discriminate (i) samples of *A. m. caucasica* (lineage O) and (ii) samples of *A. m. capensis* and *A. m. scutellata* (both of lineage A). Overall, four main clusters representing the lineages A, C, M and O can be observed (Fig. 2a,b), providing a quite high discrimination power of the 97 selected SNPs, as also already suggested^29^.

Figure 2c shows the positioning of the 61 honey bee and honey derived samples analysed with the GBS assay, in which, on the background, is maintained the position of the same samples reported in Fig. 2a,b. *A. m. ligustica* derived samples clustered in the lineage C cloud of the MDS plot, as expected. All honeycomb samples obtained from *A. m. ligustica* that were analysed by GBS had also only the C1 mtDNA mitotype, as obtained using the methods described by Utzeri et al.^43,44^. Two samples out of three of *A. m. mellifera* Ponente Ligure overlapped the lineage M spots of the MDS plot whereas the third sample was closer to the lineage C spots, as it might be derived from an admixed honey bee population. All these three *A. m. mellifera* samples had the C1 mitotype, suggesting that introgression from *A. m. ligustica* occurred in the honey bee populations that produced the analysed honey samples. *A. m. siciliana* samples were mainly included in a separated cluster, close to the C cluster. Two *A. m. siciliana* samples were positioned at the border of the C cluster, suggesting again that they might be derived from an admixed honey bee population. The mtDNA analysis confirmed for these two samples this hypothesis as both C1 and A mitotypes were detected in these two samples, whereas in all other samples, only the A mitotype was present.

### SNPs associated to calmness, gentleness and resistance to Varroa destructor

**Figure 3:**
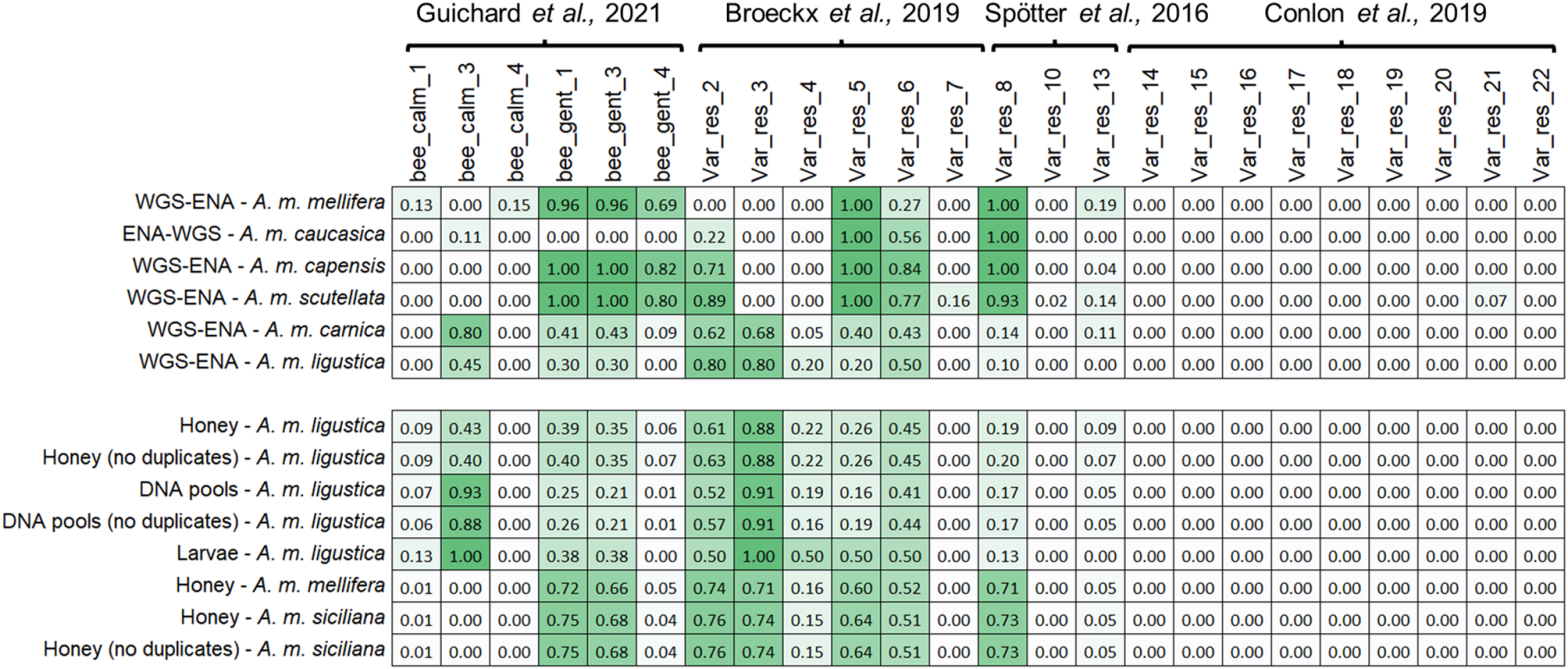
Average frequency of the alternative allele (from 0 to 1, i.e 0% to 100%) of the 24 *Apis mellifera* DNA markers linked to calmness, gentleness and varroa resistance (reference papers are provided and mentioned in the text). The colour of the box (from pale to dark green) mirrors the allele frequency. Results are reported both including and excluding duplicate samples.

The panel also included a total of 24 SNPs that, according to what was previously reported^38,40,51,52^, could be useful to provide genetic information on a few important traits of the managed honey bee colonies. Figure 3 reports a comparison of the average allele frequency of the alternative allele (according to the allele reported in the reference genome, usually associated in these cases to positive characteristics) between several *A. mellifera* subspecies, as obtained from WGS datasets retrieved from ENA, and the GBS results that we have obtained in all honeycomb samples from *A. m. ligustica*, the DNA pools from *A. m. ligustica* and the honey from *A. m. siciliana*.

About 33% of all markers (eight out of 24) were fixed for the reference and susceptible allele in all datasets retrieved from ENA and produced by GBS (Fig. 3). All these fixed markers on chromosome 15 were reported to be associated with mite resistance in two selected honey bee populations^40,56^. The absence of the alternative alleles in all other honey bee populations investigated might suggest that the positive alleles associated to mite resistance might be specific of the original populations involved in the reported studies^40,56^. Other three markers (bee_calm_4, Var_res_7 and Var_res_10) were fixed for the reference allele in all GBS datasets (Fig. 3). These markers were also fixed in almost all WGS datasets, apart in one subspecies among the investigated ones (*A. m. mellifera* for the first SNP and *A. m. scutellata* for the other two SNPs).

Similar to what reported for all SNPs, allele frequencies obtained from honey samples of *A. m. ligustica* and allele frequencies obtained from DNA pools of *A. m. ligustica* were highly correlated, with only one outlier SNP (bee_calm_3) (Supplementary Fig. S2). This could be probably due to a bias in one or few pools because allele frequencies obtained from honey were almost identical to what obtained from the WGS datasets of *A. m. ligustica* retrieved from ENA (AF = 0.43 vs AF = 0.45).

## Discussion

The availability of the honey bee reference nuclear genome^57,58^ and the production of WGS datasets from different honey bee subspecies have made it possible to identify specific population genetic footprints (e.g. SNPs and other variants) that, in turn, can be used to distinguish and allocate *A. mellifera* subspecies^18,19,26–30,32,37^. A few SNP panels have been already proposed and applied for this purpose to establish monitoring programs of the integrity of autochthonous honey bees or to evaluate the effect of conservation programs on these genetic resources^8,19,26,27,29,30^. Other genomic studies in honey bees have started the dissection of the genetic factors affecting economically relevant traits of the managed honey bee populations and a few markers have been already considered in selection programs^33,38,40,56^. These approaches usually genotyped individual honey bees. Therefore, to obtain whole colony genomic information, several individual honey bees might be analysed, with an approximation of the colony information derived by the sampling of the bees and with an increased genotyping cost, related to the number of bees genotyped.

As already reported in a few studies^40,42,45,47^, the honey, which contains the DNA of the honey bees that produced it (i.e. thousands of bees of the colony), is an easily accessible source of *A. mellifera* population genomic information that, in turn, can be useful for several applications and analyses. The easiest and simplest ways to take advantage from these honey bee traces rely on the informativity of the mtDNA of different lineages. For example, the analysis of the honey bee mtDNA from honey DNA has been proposed as tools useful to authenticate the entomological origin of this food matrix^43,44^, the analysis of hundreds of honey samples produced over all Italy has provided an updated distribution map of honey bee mtDNA lineages in this peninsula and in its two major islands, Sardinia and Sicily^45^. This information should be integrated with nuclear genome data that could better capture information on the different subspecies and, potentially, the level of admixture between subspecies. Starting from honey, however, the analysis of the honey bee nuclear genome is more complex. As mentioned, honey DNA may contain DNA traces from thousands of honey bees of the colony from which the honey derives. An untargeted approach based on whole honey DNA shot gun sequencing, even feasible, needs (i) a very high sequencing depth to be sure to obtain enough honey bee sequence information to capture genomic information representative of the colony and (ii) properly designed bioinformatic pipelines to filter out non-honey bee sequenced reads and call the relevant variants from the *A. mellifera* nuclear genome^42^. The sequencing cost and computation time and effort with this approach are high and cannot be applied routinely and on many samples. Therefore, a targeted approach designed with the aim to retrieve pre-defined sequence information from honey DNA is much more feasible. We already reported a targeted analysis of the variability in the *A. mellifera csd* gene starting from honey DNA to obtain allele frequency information for this gene at the colony level^47^. Therefore, a similar approach might be possible even for SNP genotyping, as demonstrated in this work. This study, therefore, constitutes the first example of the use of eDNA to routinely retrieve multi-marker population genomic information of *A. mellifera*.

In this study we developed a targeted SNP assay based on GBS that was useful to obtain *A. mellifera* nuclear genome information, representative of the whole colony, using honey as source of honey bee DNA. The method can estimate allele frequencies from environmental samples and complex matrices where more than one individual honey bee contributed to leave traces (honey as described in this study) and also from artificially constructed honey bee DNA pools. GBS, however, can also be applied to single individual honey bee DNA.

As expected, sequencing performances were better on DNA directly extracted from honey bees than on DNA extracted from honey, due to the higher level of DNA degradation of this food matrix than that of honey bees, which were properly sampled for this purpose^49,59^. Honey undifferentiated samples usually had a lower number of reads and a lower depth of sequencing than the honey retrieved directly from honeycombs, which again might be due to the lower level of degradation of the DNA in fresh honey samples that were not processed or stored for long time, as it might have happened for undifferentiated honey. This problem is also evidenced by the lower SNP call rate that all honey samples had if compared to the genotyping results obtained from honey bee DNA samples (both from individual larvae and DNA pools). As expected, again, genotyping concordance, as estimated by re-analysing twice the same samples (starting from the beginning of the analytical process: the DNA extraction step), was lower in honey samples than in DNA pools from honey bees. It is also clear that estimation of allele frequencies from different aliquots of honey could be eventually affected by unequal contributions of honey bee traces in the two aliquots. Despite that, correlation in duplicate honey samples was always higher than 90% and SNP call rate was for most markers ≥ 90%. Average depth of sequencing remained enough for the genotyping of different targeted alleles and, in addition, made it possible to identify other variants. These linked polymorphic sites could be eventually integrated in subsequent population genomic analyses after having evaluated their linkage disequilibrium level with the close targeted SNPs and their informativity across honey bee subspecies and populations.

Different levels of information were produced or retrieved for *A. m. ligustica*. Therefore, we also estimated if population genomic data derived from the selected SNPs could be in agreement in the different sources of information, which included: (i) individual honey bee larvae analysed separately and then considered together (that provided information on a total of four individual diploid workers, for a total of eight allele copies at each SNP), (ii) DNA pools constructed each from 35 worker bees (which provided a total combined information of 70 allele copies at each SNP), (iii) honeycomb honey DNA which provided an undefined number of allele copies (but probably on the order of thousands of copies), considering that thousands of worker bees of a colony might have contributed to produce the honey and (iv) publicly available WGS datasets obtained by other studies that are not related to our work and that provided a total of combined information from 20 allele copies at each SNPs. Correlation was always very high at all levels, with the highest correlations that was obtained between the DNA pools and the honeycomb samples, which are based on the highest number of allele copies. Some sampling biases might be due to the low number of allele copies considered in the comparative analyses. A few outlier SNPs were also identified, but their effect was diluted over the information retrieved from a large panel of SNPs.

Overall information obtained from the ancestry informative SNPs summarized in MDS plots further demonstrated the possibility to use GBS data produced from honey DNA to properly detect the honey bee subspecies from which the honey was produced. All honey samples produced by *A. m. ligustica* clustered in the expected cloud (Fig. 2) that was also generated combining different sources of information produced by others (i.e. publicly available WGS datasets and results from Henriques et al.^29^). These results were also confirmed by the same position in the cloud that was obtained by the other matrices derived from *A. m. ligustica* (i.e. DNA pools and individual bees). All these honey samples had only the C1 mitotype confirming that both at the nuclear genome level and at the mtDNA level the genetic footprint that was possible to retrieve using genomic approaches was concordant. A few honey samples derived from *A. m. siciliana* and *A. m. mellifera* clustered in intermediate positions or just at the border of the *A. m. ligustica* cluster (Fig. 2), suggesting that they might be derived from admixed honey bee populations, as also expected from the mtDNA information. Other studies suggested that *A. m. mellifera* populations in Liguria and *A. m. siciliana* populations experienced admixture events from *A. m. ligustica*^30,45,60,61^.

As the classical methods used to identify *A. mellifera* subspecies rely on morphometric analyses of the worker bees^3,48^, by definition, these approaches cannot be applied to the honey that lose the link with the colony or the apiary that produced it. Therefore, other methods to authenticate the entomological origin (i.e. honey bee subspecies) of the honey are needed. Based on the results we obtained in this study, the designed GBS approach can be useful to authenticate the entomological origin of the honey directly using information of *A. mellifera* nuclear genome markers. This method is complementary to the methods based only on mtDNA-designed assays that we and other already proposed^43,44,62^ and add another level of information for this purpose, filling the gap that might be derived from undetected admixtures between different subspecies that might not be evident from the maternal information retrieved from the mtDNA. This method can be also used to obtain population genomic information that could be useful to monitor the integrity of autochthonous honey bee genetic resources starting from a food matrix, that in several cases, might be simpler and more cost effective to be sampled than individual honey bees. The unbiased population genomic information contained in honey samples might be also useful to design novel and alternative programs aimed at monitoring *A. mellifera* subspecies genetic integrity with a lower genotyping cost than that of genotyping individual honey bees which might not be representative of the whole colony population when just one or few bees per colony are analysed.

The usefulness of the GBS approach that we tested was also demonstrated by the possibility to retrieve additional information on relevant markers that other studies reported to be associated with important characteristics of the managed colonies. The overall population genomic information that can be retrieved from honey samples, again, can easily provide a quick and cost-effective picture of the distribution and diffusion of relevant polymorphic sites across populations and eventually to consider the usefulness of these variants in queen breeding programs.

The flexibility of the GBS approach can make it possible to easily change SNPs (i.e. eliminate or add) in the described panel. Some SNPs might not be useful as, from the reported results, their informativity is poor (for example some are fixed in all populations) or cannot be successfully genotyped in all honey samples and might be more sensitive to the problem of DNA degradation, that can be critical for the analysis of honey DNA. Other SNPs, more informative than those included in this panel, can be added to increase the discrimination power and their usefulness to differentiate, for example, *A. m. ligustica* from *A. m. carnica* that could not be easily separated with this panel.

This study demonstrated, as proof of concept, that honey can be easily analysed to retrieve *A. mellifera* genotyping information from nuclear genome polymorphic sites for downstream applications, including the authentication of the entomological origin of this food matrix. Honey bee colonies are described as superorganisms that however are each composed by thousands of different bees, all genetically different. A genomic analysis of the honey produced by the colony, which gather together DNA traces of many of the individually different bees that constitute the colony itself, makes it possible to obtain in just a single and simple assay valuable information that well represents the superorganism.

## Supplementary Information

Supplementary Information 1.

Supplementary Information 2.

## Data Availability

The 61 sequencing datasets generated and analysed during the current study are available in the EMBL-EBI European Nucleotide Archive (ENA) repository (http://www.ebi.ac.uk/ena) under the study accession PRJEB55169 (https://www.ebi.ac.uk/ena/browser/view/PRJEB55169; samples ERS12561680-ERS12561740; runs ERR10031183-ERR10031243).
