# Supplementary material for: A genotyping by sequencing approach can disclose Apis mellifera population genomic information contained in honey environmental DNA
Source: Sci Rep. 2022 Nov 15;12:19541. doi: 10.1038/s41598-022-24101-z (PMC9666642; doi:10.1038/s41598-022-24101-z)
Supplement: Supplementary file 1 — Supplementary Information 1. [file 41598_2022_24101_MOESM1_ESM.docx]

**Supplementary information**

**A genotyping by sequencing approach can disclose *Apis mellifera* population genomic information contained in honey environmental DNA**

Samuele Bovo^1^, Valerio Joe Utzeri^1^, Anisa Ribani^1^, Valeria Taurisano^1^, Giuseppina Schiavo^1^, and Luca Fontanesi^1*^

^1^ Department of Agricultural and Food Sciences, Division of Animal Sciences, University of Bologna, Viale Giuseppe Fanin 46, 40127 Bologna, Italy

* Corresponding author

E-mail: [luca.fontanesi@unibo.it](mailto:luca.fontanesi@unibo.it) (LF)

**Supplementary Table S1**. Details about the 61 samples/specimens analysed by genotyping by sequencing. Sequencing statistics are included.

*See the Excel file* ***Supplementary Table S1****.*

**Supplementary Table S2**. Details about the 121 *A. mellifera* SNPs included in the panel.

*See the Excel file* ***Supplementary Table S2****.*

**Supplementary Table S3**. Details of the additional 450 honey bee samples genotyped by Henriques et al. [29] and of the 161 WGS datasets retrieved from the ENA resource.

*See the Excel file* ***Supplementary Table S3****.*

**Supplementary Table S4**. Additional *Apis mellifera* DNA variants (no. 152) identified in the amplicons of the targeted samples.

*See the Excel file* ***Supplementary Table S4****.*

**Supplementary Table S****5**. Frequency of the alternative allele (ranging from 0 to 100% for honey or DNA pools; 0%, 50% or 100% for single larvae) of the 121 *A. mellifera* SNPs included in the panel.

*See the Excel file* ***Supplementary Table S5****.*

**Supplementary Table S6**. Correlation coefficients estimated by using the average allele frequencies of whole SNP set (no. 121). Upper triangular matrix: Spearman's Rank correlation coefficient; lower triangular matrix: Pearson’s correlation coefficient.

| ***A. m. ligustica* (121 SNPs)** | **WGS-ENA** | **Honey** | **Honey**  **(no duplicates)** | **Honey bee D**NA **pools** | **Honey bee D**NA **pools (no duplicates)** | **Larvae** |
| --- | --- | --- | --- | --- | --- | --- |
| **WGS-ENA** |  | 0.84 | 0.83 | 0.83 | 0.82 | 0.76 |
| **Honey** | 0.96 |  | 1.00 | 0.91 | 0.92 | 0.83 |
| **Honey**  **(no duplicates)** | 0.96 | 1.00 |  | 0.91 | 0.91 | 0.83 |
| **Honey bee D**NA **pools** | 0.95 | 0.96 | 0.96 |  | 1.00 | 0.81 |
| **Honey bee D**NA **pools (no duplicates)** | 0.95 | 0.97 | 0.97 | 1.00 |  | 0.82 |
| **Larvae** | 0.92 | 0.93 | 0.93 | 0.93 | 0.93 |  |

**Supplementary Table S7**. Correlation coefficients estimated by using the average allele frequencies of the ancestry informative markers (no. 97). Upper triangular matrix: Spearman's Rank correlation coefficient; lower triangular matrix: Pearson’s correlation coefficient.

| ***A. m. ligustica***  **(97 SNPs)** | **WGS-ENA** | **Honey** | **Honey**  **(no duplicates)** | **Honey bee D**NA **pools** | **Honey bee D**NA **pools (no duplicates)** | **Larvae** |
| --- | --- | --- | --- | --- | --- | --- |
| **WGS-ENA** |  | 0.96 | 0.95 | 0.95 | 0.95 | 0.93 |
| **Honey** | 0.96 |  | 1.00 | 0.97 | 0.97 | 0.94 |
| **Honey**  **(no duplicates)** | 0.95 | 1.00 |  | 0.97 | 0.97 | 0.94 |
| **Honey bee D**NA **pools** | 0.95 | 0.97 | 0.97 |  | 1.00 | 0.93 |
| **Honey bee D**NA **pools**  **(no duplicates)** | 0.95 | 0.97 | 0.97 | 1.00 |  | 0.93 |
| **Larvae** | 0.93 | 0.94 | 0.94 | 0.93 | 0.93 |  |

**Supplementary Table S8**. Correlation coefficients estimated by using the average allele frequencies of the SNPs associated to calmness, gentleness and resistance to *Varroa destructor* (no. 24). Upper triangular matrix: Spearman's Rank correlation coefficient; lower triangular matrix: Pearson’s correlation coefficient.

| ***A. m. ligustica***  **(24 SNPs)** | **WGS-ENA** | **Honey** | **Honey**  **(no duplicates)** | **Honey bee D**NA **pools** | **Honey bee D**NA **pools (no duplicates)** | **Larvae** |
| --- | --- | --- | --- | --- | --- | --- |
| **WGS-ENA** |  | 0.93 | 0.93 | 0.92 | 0.92 | 0.94 |
| **Honey** | 0.97 |  | 1.00 | 0.99 | 1.00 | 0.93 |
| **Honey**  **(no duplicates)** | 0.97 | 1.00 |  | 0.99 | 0.99 | 0.93 |
| **Honey bee D**NA **pools** | 0.89 | 0.90 | 0.89 |  | 1.00 | 0.94 |
| **Honey bee D**NA **pools**  **(no duplicates)** | 0.92 | 0.92 | 0.91 | 1.00 |  | 0.94 |
| **Larvae** | 0.87 | 0.90 | 0.89 | 0.95 | 0.95 |  |

**Supplementary Table S9**. Matrix of size 672 samples × 97 SNPs storing the frequency of the alternative allele (ranging from 0 to 100% for honey or DNA pools; 0%, 50% or 100% for single larvae) for the 97 ancestry informative markers in the whole dataset.

*See the Excel file* ***Supplementary Table S9****.*

**Supplementary Fig. S1**. Scatter plots based on frequencies of the alternative allele of the 121 *Apis mellifera* DNA marker targeted by the panel. Data are presented for the duplicate samples. Data are presented including/excluding duplicate samples. The intra-class correlation coefficient (ICC) is reported.


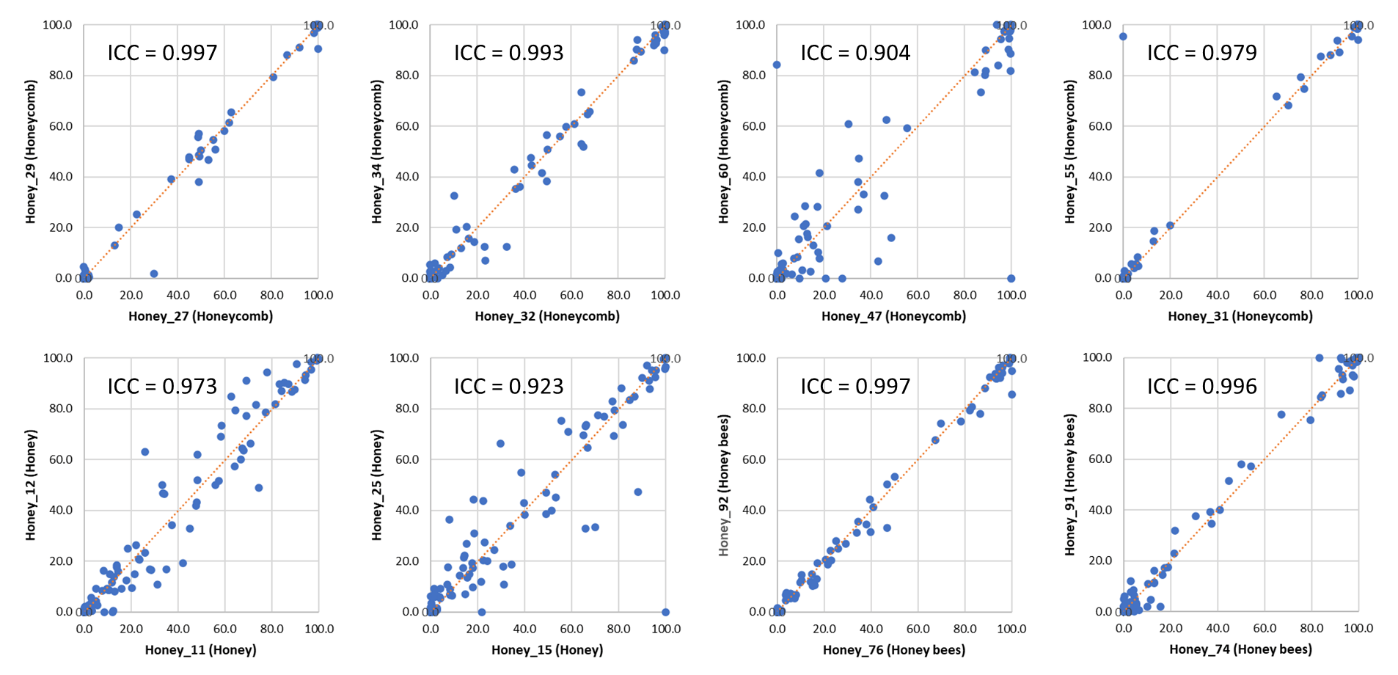


**Supplementary Fig. S2.** Scatter plots based on frequencies of the alternative allele of the 121 targeted *Apis mellifera* DNA markers. Data are related to the *A. m. ligustica* larvae samples compared to the remaining datasets. Data are presented including/excluding duplicate samples.


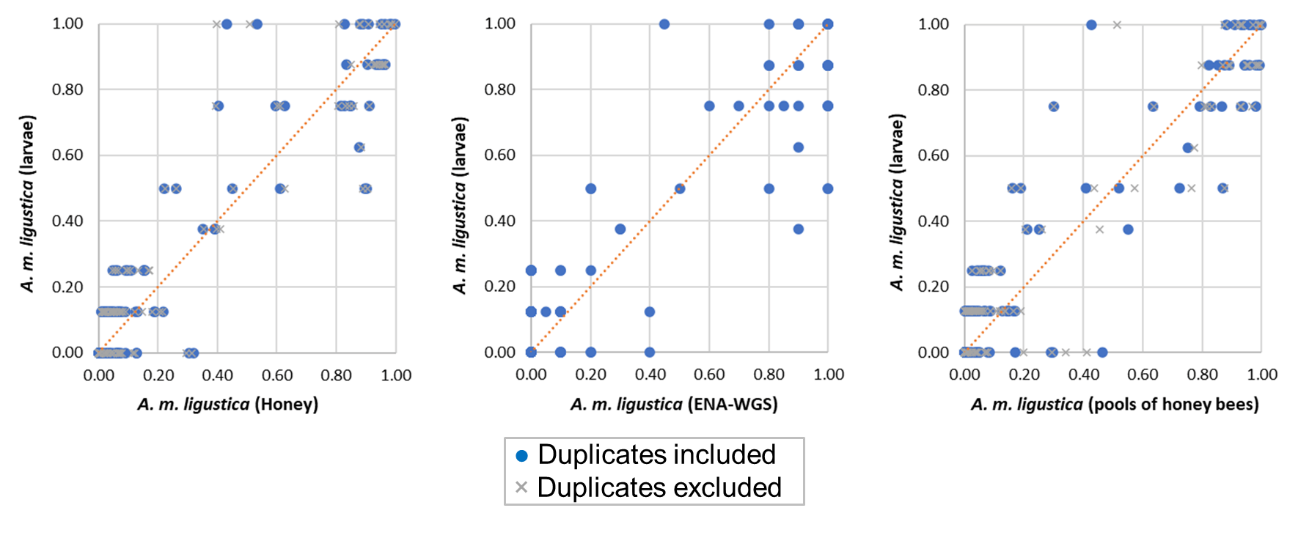


**Supplementary Fig. S3.** Scatter plots based on frequencies of the alternative allele of the 24 DNA markers linked to calmness, gentleness and varroa resistance targeted by the panel. Data are related to the *A. m. ligustica* datasets and samples for which multiple levels of information were available. Data are presented including/excluding duplicate samples.

**
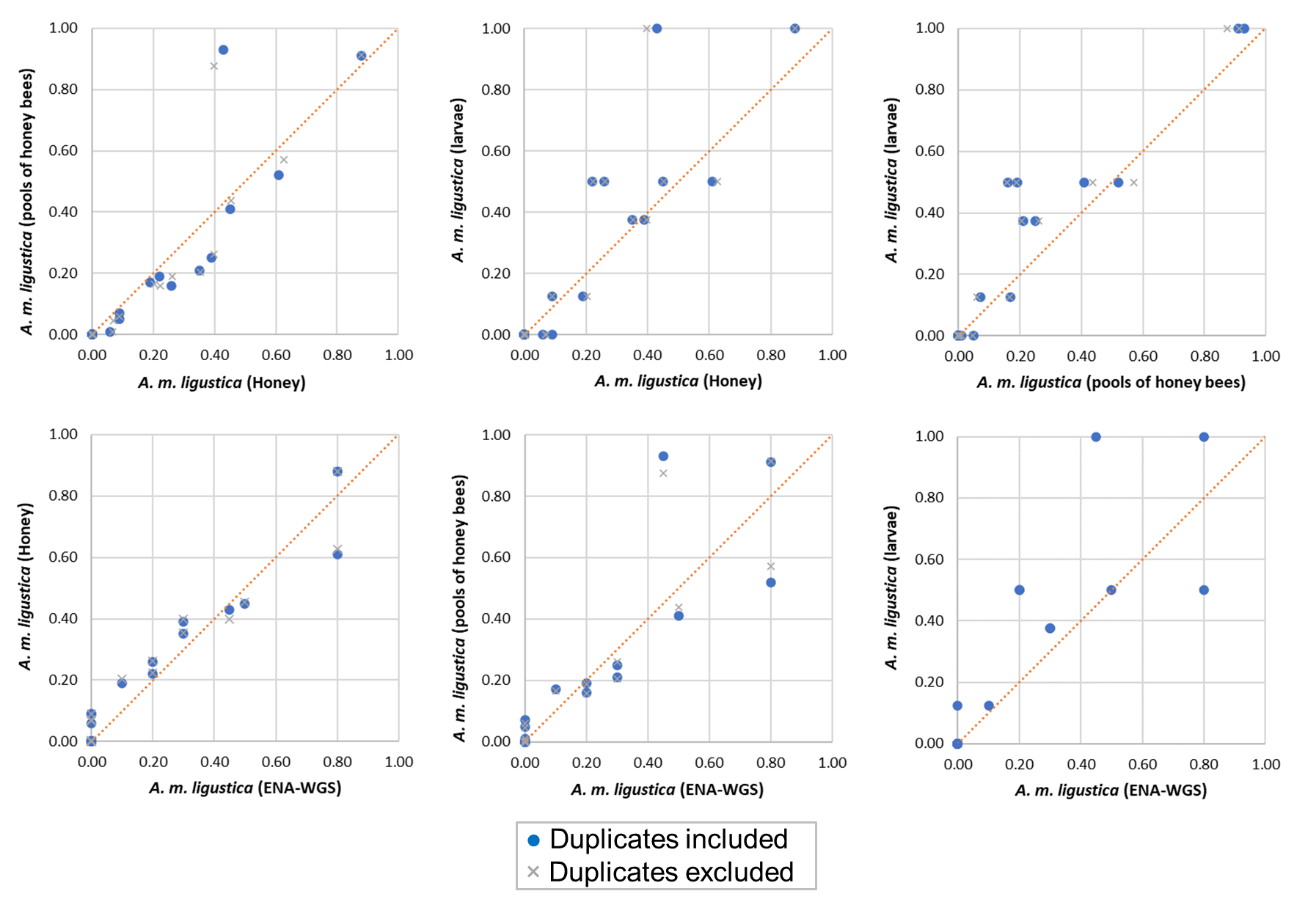
**
